# Induction of Protective Immune Responses Against *Schistosomiasis haematobium* in Hamsters and Mice Using Cysteine Peptidase-Based Vaccine

**DOI:** 10.3389/fimmu.2015.00130

**Published:** 2015-03-23

**Authors:** Hatem Tallima, John P. Dalton, Rashika El Ridi

**Affiliations:** ^1^Zoology Department, Faculty of Science, Cairo University, Giza, Egypt; ^2^Medical Biology Centre, School of Biological Sciences, Queen’s University Belfast, Belfast, UK

**Keywords:** *Schistosoma haematobium*, schistosomiasis vaccine, cysteine peptidases, papain, cathepsins, type 1 and 2 immune responses, larval excretory–secretory products

## Abstract

One of the major lessons we learned from the radiation-attenuated cercariae vaccine studies is that protective immunity against schistosomiasis is dependent on the induction of T helper (Th)1-/Th2-related immune responses. Since most schistosome larval and adult-worm-derived molecules used for vaccination uniformly induce a polarized Th1 response, it was essential to include a type 2 immune response-inducing molecule, such as cysteine peptidases, in the vaccine formula. Here, we demonstrate that a single subcutaneous injection of Syrian hamsters with 200 μg active papain, 1 h before percutaneous exposure to 150 cercariae of *Schistosoma haematobium*, led to highly significant (*P* < 0.005) reduction of >50% in worm burden and worm egg counts in intestine. Immunization of hamsters with 20 μg recombinant glyceraldehyde 3-phosphate dehydrogenase (rSG3PDH) and 20 μg 2-cys peroxiredoxin-derived peptide in a multiple antigen peptide construct (PRX MAP) together with papain (20 μg/hamster), as adjuvant led to considerable (64%) protection against challenge *S. haematobium* infection, similar to the levels reported with irradiated cercariae. Cysteine peptidases-based vaccination was also effective in protecting outbred mice against a percutaneous challenge infection with *S. haematobium* cercariae. In two experiments, a mixture of *Schistosoma mansoni* cathepsin B1 (SmCB1) and *Fasciola hepatica* cathepsin L1 (FhCL1) led to highly significant (*P* < 0.005) reduction of 70% in challenge *S. haematobium* worm burden and 60% reduction in liver egg counts. Mice vaccinated with SmCB1/FhCL1/rSG3PDH mixture and challenged with *S. haematobium* cercariae 3 weeks after the second immunization displayed highly significant (*P* < 0.005) reduction of 72% in challenge worm burden and no eggs in liver of 8–10 mice/group, as compared to unimmunized mice, associated with production of a mixture of type 1- and type 2-related cytokines and antibody responses.

## Introduction

Schistosomiasis is a debilitating parasitic disease that affects humans in 74 countries, mainly in the Middle East, sub-Saharan Africa, South America, and some regions of the Philippines, China, and Indonesia. Two species, *Schistosoma mansoni* and *Schistosoma haematobium*, are responsible for the majority of human infections. As a result of the insensitivity and unreliability of current diagnostic techniques and the paucity of sound epidemiological surveys, it is not clear whether the number of active *Schistosoma* infections is 209 (1), 230 (2), 252 (3), or 391–587 (4) million people worldwide. People infected with schistosomes react intensely to antigens derived from the huge numbers of parasite eggs that have failed to escape to the exterior via feces (*S. mansoni*) or urine (*S. haematobium*), and are trapped in the host tissues. These intense immunological reactions lead to fibrosis and dysfunction of the affected organs, namely liver, gut, and urinary bladder (1–5).

A single anti-schistosome drug, praziquantel (PZQ), is readily available. Despite its low cost and self-limiting side-reactions, the drug has only been offered to less than 13% of the target population (1). Innumerable persons are left untreated, suffering long-term disabilities and exacerbation of co-infections (1–6). Praziquantel is highly effective in treatment of light and moderate infections. However, in areas of high endemicity and transmission and/or intensive PZQ mass administration, PZQ cure rates are almost negligible [(7) and references therein]. A schistosomiasis vaccine could protect up to 600–780 million individuals, mostly children, living in endemic regions at risk of the infection. Articles in this topic and elsewhere have duly reported on the history and fate of a number of candidates and potential vaccine antigens, of which very few have shown satisfactory efficacy and none has reached the commercial level (8–10).

One of the main reasons hindering the development of a vaccine against schistosomiasis is the entrenched dogma stating protection is dependent on the generation of type 1 immune responses. This belief was based on preponderance of interferon-gamma (IFN-γ) released by bronchoalveolar leukocytes, total lung tissue, and lung-draining lymph nodes in radiation-attenuated (RA) cercariae-vaccinated mice (11, 12). Lung schistosomula-derived antigens seeping in lung tissues or released from extravasated dying larvae expectedly induce preponderant type 1 immune responses [(13, 14) and references therein]. Yet, these immune responses might be irrelevant to parasite attrition, as it must be reiterated healthy schistosomes are exclusively intravascular and may not be directly affected by the immune events in lung alveoli, parenchyma, or draining lymph nodes. More importantly, several studies using knockout mice conclusively demonstrated that the optimal protection in the RA vaccine model is dependent on the induction of both type-1 and type-2-associated immune responses (15–17).

We have well-learned the lessons of the successful RA vaccine model and thought it is imperative to use type 2-, not type 1-inducing cytokines or molecules as adjuvants to the schistosome-derived antigens used for vaccination (14). The highly significant (*P* < 0.0001) and reproducible protection against challenge *S. mansoni* worms achieved in mice, immunized with larval antigens derived from excretory–secretory products (ESP), namely recombinant glyceraldehyde 3-phosphate dehydrogenase (rSG3PDH) and 2-cys peroxiredoxin-derived peptide in a multiple antigen peptide construct (PRX MAP) in conjunction with papain, interleukin (IL)-25, IL-33, or thymic stromal lymphopoietin (TSLP), supported our belief. Our proposal was particularly strengthened by the significant (*P* < 0.02) levels of protection obtained following immunization with papain, IL-25, or IL-33 alone [(18, 19) and references therein]. Therefore, we felt it was important to examine whether this approach could be applied to *S. haematobium*, and examined immunological and parasitological parameters in hamsters immunized with papain alone, or papain in conjunction with rSG3PDH and PRX MAP.

Since papain, IL-25, IL-33, or TSLP may not be readily used for human vaccination, we resolved the issue by replacing these type 2-inducing molecules by parasite-derived cysteine peptidase, namely *S. mansoni* cathepsin B1 (SmCB1). Immunization of outbred mice with SmCB1 alone generated a polarized type 2 immune response environment that was associated with highly significant (*P* < 0.0001) reduction of 83% of *S. mansoni* challenge worm burden; this supported our hypothesis stating that *S*. *mansoni* larvae will almost all succumb if met by a type 2 cytokine environment (18–21). To further improve the vaccine efficacy, we included another cysteine peptidase, *Fasciola hepatica* cathepsin L with the aim of inducing the production of anti-cathepsin L antibodies that would neutralize the *S. mansoni* homologous enzyme and inhibit its function. The highest level of worm burden reduction and decrease in worm egg counts in liver and small intestine of outbred mice were achieved when this peptide formulation was combined with rSG3PDH (22–25). It is important to note that SG3PDH is a larval and adult worm ESP (26), documented to be also associated with the larval surface membrane (22), and to induce polarized type 1 and type 17 immune responses (19).

Therefore, we proposed a novel schistosome cysteine peptidase-based formula that fulfills all requirements for an efficacious vaccine for schistosomiasis (24, 25). First, two immunizations are sufficient to induce highly significant (*P* < 0.0001) and highly reproducible (eight experiments) reduction of up to 66% in *S. mansoni* worm burden and egg counts in host liver and intestine. Second, the vaccine is adjuvant/chemical free, bypassing the insurmountable obstacle of adjuvant use in pre- and clinical trials in humans. Third, vaccine-induced protection is associated with generation of both type 1 and type 2 cytokines-related immune responses. Fourth, the vaccine was entirely safe in outbred mice and did not induce IgE antibodies or any adverse reaction during immunization and after challenge.

To proceed forward with and efficacious vaccine formula against *S. mansoni*, it is important that we demonstrate that these approaches apply to *S. haematobium* and *S. japonicum*. Indeed, most vaccine strategies applied to date have not shown cross-species efficacy. Moreover, vaccine studies in the *S. haematobium* model are rather rare and, thus, the present study represents an addition to this neglected field. Accordingly, we, herein, investigated whether our vaccine formulation of functional cysteine peptides without the addition of a chemical adjuvant is also effective in protecting mice against a challenge infection with *S. haematobium*.

## Materials and Methods

### Ethics statement

All animal experiments were performed following the recommendations of the current edition of the Guide for the Care and Use of Laboratory Animals, Institute of Laboratory Animal Resources, National Research Council, USA, and were approved by the Institutional Animal Care and Use Committee (IACUC) of the Faculty of Science, Cairo University, permit number CUFS F PHY 21 14.

### Animals and parasites

Female Syrian hamsters (*Mesocricetus auratus*) and CD1 mice were raised at the Schistosome Biological Materials Supply Program, Theodore Bilharz Research Institute (SBSP/TBRI), Giza, Egypt, and maintained throughout experimentation at the animal facility of the Zoology Department, Faculty of Science, Cairo University. Cercariae of an Egyptian strain of *S. haematobium* were obtained from SBSP/TBRI, and used for infection immediately after shedding from *Bulinus truncatus* snails.

### Papain and immunogens

Papain from *Carica papaya* (BioChemika ≥3 units/mg) was obtained from BioChemika, and used in an active form or following inactivation by incubation in the presence of 5 μM of the irreversible inhibitor of cysteine peptidases, l-trans-epoxysuccinylleucylamide-(4-guanido)-butane (E-64, Calbiochem, San Diego, CA, USA), as described previously (24, 27). Recombinant *S. mansoni* glyceraldehyde 3-phosphate dehydrogenase (rSG3PDH) was prepared and purified to homogeneity, as described (23) and contained <0.06 Endotoxin Units/ml as judged by the Pyrogen Gel-Clot Limulus Amebocyte Lysate test. 2-Cys peroxiredoxin (28) (H-^104^RKQEISKAYGVFDE EDGNA^122^-OH)-derived peptide, showing lowest homology to the murine counterpart, was synthesized as a MAP (tetra branched multiple antigen peptide) construct (PRX MAP) and purified at AnaSpec Inc. (San Jose, CA, USA). Functionally active *S. mansoni* cathepsin B1 (SmCB1) and *F. hepatica* cathepsin L1 (FhCL1) were prepared as described (24, 29).

These *S. mansoni*-derived molecules were used for vaccination of hamsters and mice against infection with *S*. *haematobium*, as they are remarkably conserved across *S. mansoni* and *S*. *haematobium*. *S. mansoni* cathepsin B1 and SG3PDH show 94–96% identities at the amino acid level with the corresponding enzyme of *S. haematobium* (GI:68596858 and GI:685936895, respectively). *S. mansoni* 2-Cys peroxiredoxin (PRX)-derived peptide used in the MAP construct shows 84% identities and 89% similarity with the corresponding peptide of *S. haematobium* PRX (GI:685965340). *F. hepatica* cathepsin L was readily recognized by outbred mice infected with *S. mansoni*.

### Injection, infection, and analyses in hamsters

Hamsters (10 per group) were injected subcutaneously (sc) into two sites with 200 μg active papain. One hour later, injected and untreated hamsters were anesthetized, abdomen-shaved, and then exposed percutaneously using the ring method (30) to 150 *S. haematobium* cercariae.

Hamsters (10 or11 per group) were immunized, twice at 3 weeks interval, sc on one side with 20 μg active or E-64-inactivated papain, and intramuscularly on the other side with 20 μg rSG3PDH and 20 μg PRX MAP. Four weeks later, naïve and immunized hamsters were exposed to 120 cercariae of *S. haematobium* as described above.

Serum was recovered from three hamsters per group per experiment on day 14 post infection. Sera were individually assessed by enzyme-linked immunosorbent assay (ELISA) for antibody binding to soluble *S. haematobium* adult worm antigen (SAWA, 1.0 μg/well) prepared as described (31), rSG3PDH (250 ng/well), and PRX MAP (1.0 μg/well). Alkaline phosphatase (AKP)-labeled anti-hamster IgG (H + L) conjugate (Kirkegaard and Perry Laboratories, Gaithersburg, MD, USA) was diluted 1:1000. For each experiment, antibody isotypes of individual sera, diluted 1:50, for each hamster group were determined using biotin-labeled monoclonal antibodies to hamster IgG classes, IgG1, and IgG2 (Pharmingen, San Diego, CA, USA), and AKP-labeled streptavidin from Promega (Madison, WI, USA).

Worm burden and liver and intestine worm egg load in individual hamsters (6–8 per group) were evaluated 12 weeks after challenge infection (19, 24). Mean values ± SE for each group were calculated. Percent change was evaluated by the formula: % change = mean number in infected controls - mean number in infected, treated mice/mean number in infected controls × 100.

### Immunizations and infections in mice

In two experiments, mice (12–13 per group) were immunized sc at the base of the tail, twice with a 3-week interval with 10 μg SmCB1 and 10 μg FhCL1 alone or combined with rSG3PDH (10 μg/mouse). Three weeks after the second injection, untreated and immunized mice were infected percutaneously via whole body exposure to 100 ± 5 viable cercariae of *S. haematobiu*m. Spleen cells and serum were recovered from 2–3 mice per group per experiment on day 8 post infection. Worm burden and liver worm egg load were assessed in mice 12 weeks after the challenge infection as described for hamsters.

### Cytokine and humoral responses

Spleen cells (SC) were harvested on day 8 after infection with *S. haematobium* cercariae, and cultured with 0 or 5 μg/ml immunogen as described (19, 24). At 48 and 72 h of incubation, cultured SC were thawed and frozen for release of intracellular cytokines, and supernatants stored at −76°C until assayed by capture ELISA for levels of IL-4, IL-5, IL-17A, IFN-γ (ELISA MAX^TM^ Set, BioLegend), and IL-13 (DuoSet ELISA Development System, R&D Systems Europe), following the manufacturer’s instructions.

Sera were obtained from unimmunized and immunized mice 8 days following infection with cercariae of *S. haematobium*, and individually assessed by ELISA for humoral antibody titer reactivity to SAWA (1.0 μg/well) and SmCB1 (250 ng per well). Antibody isotypes in mouse sera (1:200 dilution) were analyzed using rat alkaline phosphatase-conjugated monoclonal antibodies to various mouse IgG classes (Pharmingen) and biotin-labeled monoclonal antibody to mouse IgA, and IgE (BioLegend) with sera diluted 1:25 (19, 24).

### Data analysis and statistics

All values were tested for normality. Mann–Whitney test was used to analyze the statistical significance of differences between experimental and control values and considered significant at *P* < 0.05.

## Results

### Effect of pre-treatment with the cysteine peptidase, papain on hamster humoral immune responses, and resistance to *S. haematobium*

Hamster humoral antibody binding to soluble adult worm antigens (SAWA) 14 days after exposure to 150 cercariae of *S. haematobium* was negligible, and was not significantly enhanced by pre-infection treatment with active papain (data not shown). Yet, a single papain injection 1 h before hamster exposure to *S. haematobium* cercariae led to highly significant (*P* < 0.005) decrease in total worm burden of 55%. The decrease in worm burden was observed for both male and female worms (Table 1). Hamster pre-treatment with active papain before infection did not induce a significant decrease in worm egg counts in liver. However, the decrease in intestine egg counts was highly significant (*P* < 0.002), reaching 78.1% (Table 1).

**Table 1:** **Effect of papain pre-treatment on parasitological parameters of *S. haematobium*-infected hamsters**.

Parameter	Infected controls	Active papain
Total worm burden		
Mean ± SE	48.8 ± 2.6	22.1 ± 0.9
*P* value (reduction %)		<0.005 (54.7)
Male worm burden		
Mean ± SE	30.5 ± 2.6	13.3 ± 0.8
*P* value (reduction %)		<0.005 (56.3)
Female worm burden		
Mean ± SE	17.8 ± 1.2	8.5 ± 0.7
*P* value (reduction %)		<0.005 (52.2)
Liver egg counts		
Mean ± SE	70450 ± 1646	60000 ± 5400
*P* value (reduction %)		Not significant
Intestine egg counts		
Mean ± SE	16564 ± 2210	3620 ± 616
*P* value (reduction %)		<0.002 (78.4)

*Parasitological parameters were assessed 12 weeks after infection of untreated (infection controls), or active papain-injected hamsters (7 hamsters per group). Two-tailed *P* value as assessed by the Mann–Whitney test. Reduction % = mean number of untreated hamsters – mean number of papain treated hamsters/mean number in untreated hamsters × 100*.

### Effects of the cysteine peptidase, papain as adjuvant on hamster humoral immune responses, and resistance to *S. haematobium*

**Figure 1:**
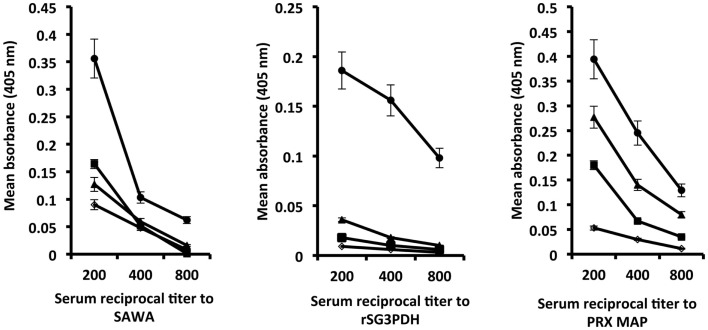
**Effect of combining rSG3PDH/PRX MAP vaccine with active papain on humoral responses of 14 day *S. haematobium*-infected hamsters**. Each point represents mean ELISA absorbance (405 nm) of sera from three individual naïve (○), infected control (■) hamsters, and hamsters immunized with inactive (▲) and active (●) papain in conjunction with rSG3PDH and PRX MAP, tested in duplicate, and the horizontal bars depict the SE around the mean.

Hamsters immunized with rSG3PDH and PRX MAP in conjunction with inactive or active papain were challenged with 120 *S. haematobium* cercariae 4 weeks later, and tested for serum antibody binding to SAWA and to the immunogens 14 days after infection. Highest antibody binding to SAWA was observed in hosts immunized with the vaccine in conjunction with active papain. The antibody binding to rSG3PDH and PRX MAP, 6 weeks after the boost immunization, is evidence for memory response to the immunogens, and was again highest in hamsters immunized with the vaccine and active papain as adjuvant (Figure 1). No immunogen-specific IgG1 antibodies were detected, while about 10% of the bound antibodies were of the IgG2 isotype (data not shown).

**Figure 2:**
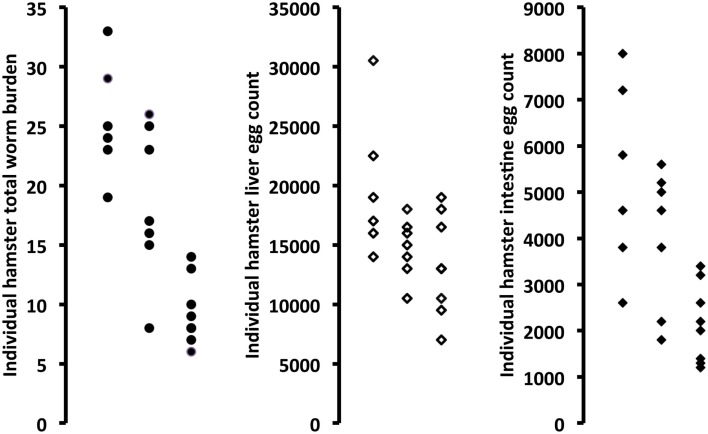
**Effect of combining rSG3PDH/PRX MAP vaccine with inactive (middle column in each panel) or active (right column in each panel) papain on parasitological parameters of *S. haematobium*-infected hamsters, as compared to unimmunized hamsters (left column in each panel)**. Each point represents parasitological parameter in individual hamsters, 12 weeks after infection with *S. haematobium* cercariae.

Active papain used as an adjuvant to rSG3PDH/PRX MAP vaccination of hamsters against *S. haematobium* led to highly significant (*P* = 0.0007) decrease of 64% in total worm burden. The decrease in worm burden was observed for both male and female worms. Inactivation of papain with E-64 almost completely eliminated its protective effect (Table 2; Figure 2). Importantly, hamster immunized with rSG3PDH/PRX MAP and active papain as adjuvant showed significant decrease in challenge *S. haematobium* worm egg counts in both liver (32.8%, *P* < 0.05) and intestine (59.4%, *P* < 0.01) (Table 2; Figure 2).

**Table 2:** **Effect of combining rSG3PDH/PRX MAP vaccine with inactive or active papain on parasitological parameters of *S. haematobium*-infected hamsters**.

Parameter	Infected controls	Inactive papain/Ag mix	Active papain/Ag mix
Total worm burden			
Mean ± SE	25.5 ± 2.0	18.7 ± 2.4	9.3 ± 2.8
*P* value (reduction %)		NS	0.0007 (63.5)
Male worm burden			
Mean ± SE	15.5 ± 1.1	11.8 ± 1.9	5.1 ± 0.6
*P* value (reduction %)		NS	0.0007 (67.1)
Female worm burden			
Mean ± SE	10.0 ± 0.9	6.8 ± 0.7	4.2 ± 0.4
*P* value (reduction %)		0.037 (32.0)	0.0007 (58.0)
Liver egg counts			
Mean ± SE	19833 ± 2438	14714 ± 937	13312 ± 1505
*P* value (reduction %)		NS	0.0481 (32.8)
Intestine egg counts			
Mean ± SE	5333 ± 840	4028 ± 566	2162 ± 301
*P* value (reduction %)		NS	0.0067 (59.4)

*Ag mix = rSG3PDH + PRX MAP. NS = not significant, as assessed by the Mann–Whitney test (two-tailed *P* value). Reduction % = mean number in unimmunized hamsters – mean number in papain/Ag mix immunized hamsters/mean number in unimmunized hamsters × 100*.

### Cysteine peptidase-based vaccine against murine *S. haematobium*

In two experiments, 9 out of 9 untreated/infected mice had 4–6 worms and 950–2400 eggs in liver. Vaccination with SmCB1/FhCL1 mixture led to highly significant (*P* < 0.005) reduction of 70% in challenge *S*. *haematobium* worm burden and 60% reduction in liver egg counts, as 5 out of 10 mice had 1–2 worms and 3 out of 10 mice showed 850–2400 eggs in liver. Mice vaccinated with SmCB1/FhCL1 and rSG3PDH displayed highly significant (*P* < 0.005) reduction of 72% in challenge worm burden and no eggs in liver of 8–10 mice/group, as compared to unimmunized mice.

**Figure 3:**
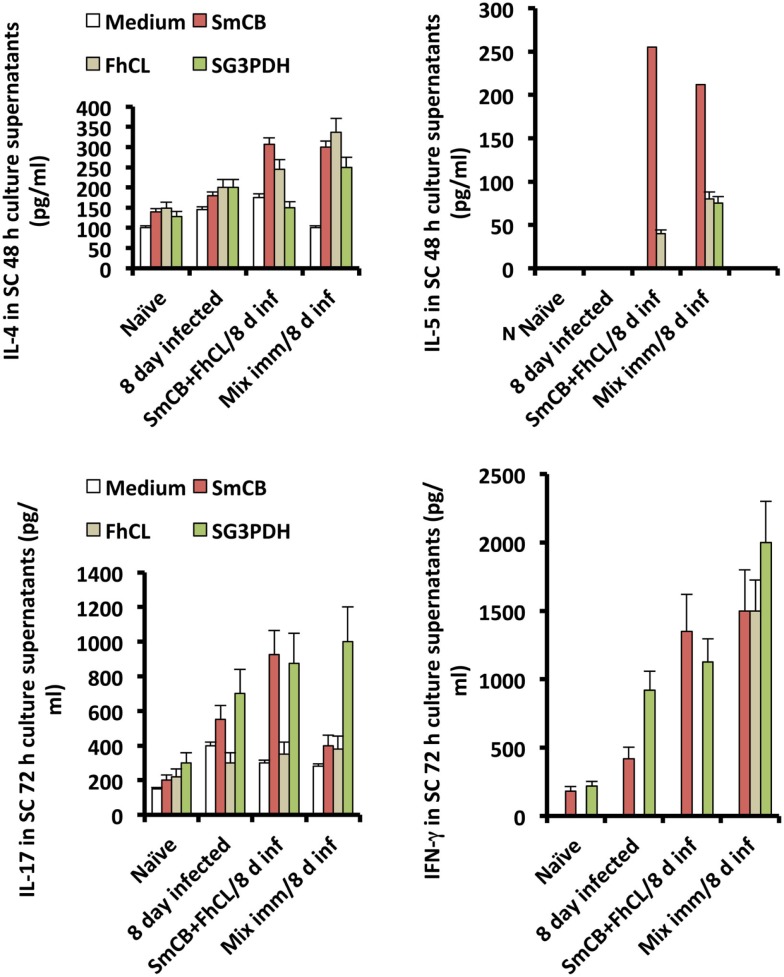
**Effects of immunization with SmCB1 + FhCL1 alone or in combination with rSG3PDH (Mix imm) on spleen cells cytokine release**. Representative of two independent experiments whereby SC obtained from naïve mice, and unimmunized or immunized mice 8 days after infection (inf) with *S. haematobium* cercariae were stimulated *in vitro* with 0 (medium) or 5 μg immunogen/well of duplicate wells. Columns represent levels of cytokines in supernatants assessed 48 (IL-4 and IL-5) and 72 h (IL-13, not shown, IL-17, and IFN-γ) later, and vertical bars denote the SE about the mean values for 2–3 mice/group.

Spleen cells obtained from unimmunized and immunized mice 8 days following *S. haematobium* infection, and stimulated *in vitro* with cysteine peptidase or rSG3PDH produced higher levels of IL-4, IL-5, IL-13, and also IL-17 and IFN-γ, compared to naïve and unimmunized infected mice, implying that cysteine peptidase-based protection against *S. haematobium* was associated with a mixture of type 1, type 2, and type 17 cytokines (Figure 3).

**Figure 4:**
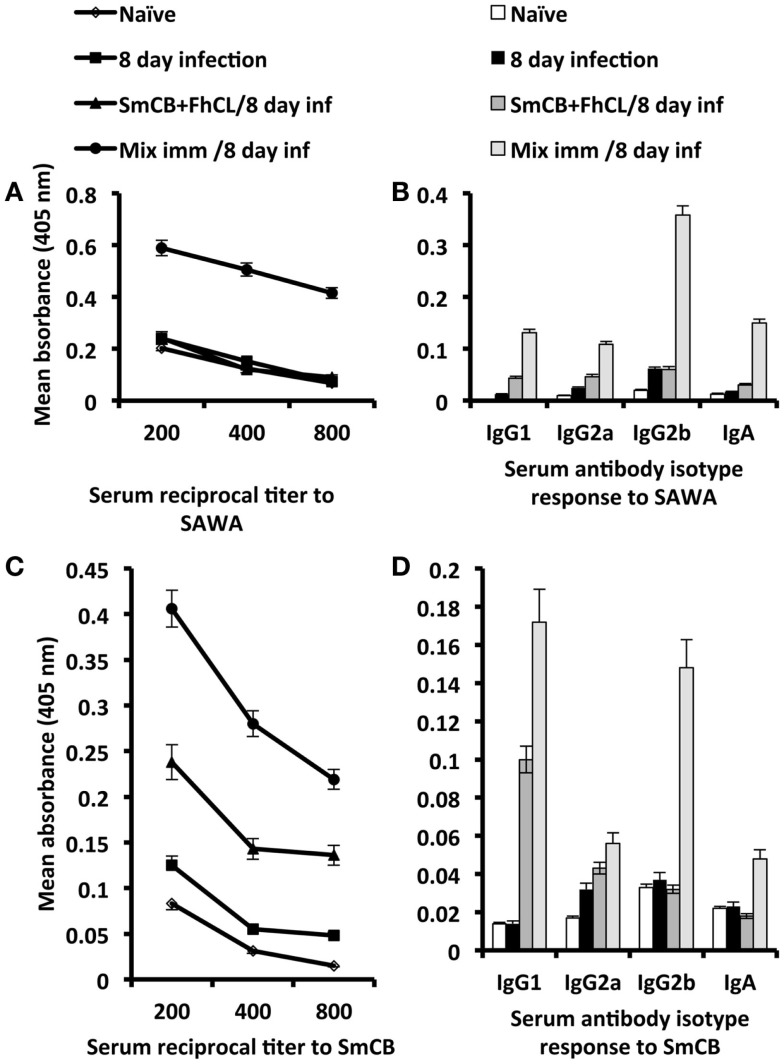
**Effects of immunization with SmCB1 + FhCL1 alone or in combination with rSG3PDH (Mix imm) on serum antibody responses**. Representative of two independent experiments whereby sera obtained from 2–3 naïve mice, and unimmunized or immunized mice 8 days after infection (inf) with *S. haematobium* cercariae were tested for antibody titer and isotype response to SAWA **(A,B)** and SmCB1 **(C,D)**. Each point or column represent mean ELISA absorbance (405 nm) of 2–3 mice tested in duplicate, and vertical bars denote the SE around the mean. No IgE antibodies were detected in sera diluted 1:25.

Serum antibody titer and isotype responses to SAWA and SmCB1 in unimmunized and immunized mice, 8 days after infection with *S. haematobium* are shown in Figure 4. Antibody responses to SAWA were observed only in mice immunized with rSG3PDH in conjunction with SmCB1 and FhCL1, and included IgG1, IgG2a, IgG2b, and IgA antibodies. No IgE antibodies were detected in sera diluted 1:25 (Figures 4A,B). High antibody responses to SmCB1 were observed in mice immunized with the cysteine peptidases and consisted predominantly of IgG1 antibodies. Serum antibody responses to SmCB1 were highest in mice immunized with the cysteine peptidases in conjunction with rSG3PDH, and included IgG1, IgG2a, IgG2b, and IgA antibodies (Figures 4C,D).

## Discussion

We have proposed that to achieve significant attrition of schistosomes during their journey in the lung blood capillaries and liver sinusoids, eosinophils and basophils must be recruited to the circulation and activated by type 2 cytokines, such as IL-4, IL-5, and IL-13, or via binding antibody-ESP complexes in the vicinity of the larvae (18–21). Worms-derived antigens induce preponderant type 1- and type 17- and limited or no type 2-related cytokines and antibodies. Accordingly, we have proposed that deviation of the host immune system toward the type 2 axis, via administering type 2 immune responses-inducing molecules, such as the cysteine peptidase, papain (32–34) before infection will lead to considerable decrease in worm burden, as compared to uninjected animals. In four consecutive experiments, 50 μg papain injected sc in outbred mice,1 h before exposure to 125 cercariae of *S*. *mansoni* consistently, and reproducibly elicited highly significant (*P* < 0.0001) reduction in worm burden of 70% ±3. The reduction was also highly significant for decrease in worm liver (*P* < 0.0001) and small intestine (*P* < 0.001) egg counts but only of approximately 50% (14).

Here, our studies with peptidases were extended to determine the validity of this approach for protection in the hamster model of *S. haematobium* (Table 1). We found that papain treatment prior to challenge infection caused a reduction in *S. haematobium* worm burden in hamsters which was not associated with significant humoral responses. We are currently exploring the molecular mechanism by which pre-treatment with active papain leads to such highly significant (*P* < 0.005) decrease in total, male and female worm burden. Second, while the reductions in intestine egg counts were considerable in papain-injected hamsters, there was no decrease in liver egg counts; this could suggest elevated fecundity in the surviving worms, perhaps due to increased levels of type 2 cytokines which has been reported to correlate with schistosome increased egg production (24, 35).

Vaccination of hamsters with our candidate vaccine mixture, rSG3PDH, and PRX MAP, in conjunction with papain led to highly significant (*P* = 0.0007) decrease of 64% in *S*. *haematobium* challenge worm burden provided using active not E-64-inactivated papain. These findings extend and confirm results recently obtained in mice immunized with the antigen mixture in conjunction with active papain and challenged with *S. mansoni*. It is of importance to note that active, but not inactivated papain, helped to generate immunogens-specific humoral antibody response that appeared to be essential for protection and decrease in worm egg load in liver. These findings suggest that the key to papain-mediated protective effect may be induction of active enzyme activity-dependent long-lived antibody-secreting cells, similarly to the proteases, natterins from the venom of *Thalassophryne nattereri* fish (36, 37).

Since papain derived from the plant *Carica papaya* could not be used for human vaccination, we sought to replace this by the *S*. *mansoni* cysteine peptidase, SmCB1, and the *F. hepatica* cathepsin L, both members of the papain-like peptidase family. As reported previously, immunization of outbred mice with SmCB1 + FhCL1 + rSG3PDH elicited highly significant (*P* < 0.0001) decrease of about 66% in challenge *S. mansoni* worm burden and worm egg counts in liver and intestine, distinctly higher than for SmCB1 and FhCL1, without rSG3PDH (24, 25). These results were reproduced here since by demonstrating protection of mice against challenge *S. haematobium* with similar formulations. Protection against murine *S. haematobium* appeared to be associated with induction of Th1, Th17, and Th2 cytokines and antibody responses, corroborating our suggestion that the generation of type 2-related immune responses is important in the design of an effective schistosomiasis vaccine (18–21, 24, 25).

The cysteine peptidases used in the study are purified as zymogens, which are stabilized by their propeptide segments. The schistosome and fasciola peptidases have been shown previously by Dalton and colleagues (38–41) to be extremely stable and not readily susceptible to breakdown. Jilkova et al. (42) recently described how the propeptide of SmCB1 can stabilize the enzyme and resist auto-processing, and suggested that the enzyme can be activated when delivered *in vivo* by tissue glycoaminoglycans. Therefore, it is possible that following injection of the cysteine proteases, these become activated to mature enzymes by interaction with glycoaminoglycans under the skin.

Concerns may be raised for the future use of cysteine peptidases for vaccination of humans because of their potential to induce IgE antibodies. However, the cysteine peptidases used in the vaccine formula consistently failed to elicit production of IgE in mice despite booster immunizations with SmCB1 and FhCL1 alone or combined (this study and 24). Additionally, serum antibodies of *S. mansoni*-infected humans that bound to SmCB1 and FhCL1 were found to be essentially of the IgG and IgA isotype (24, 43, 44). Nonetheless, helminth cysteine peptidases-induced IgE may be irrelevant for children in rural areas of the developing world, to whom the vaccine is intended, as these usually harbor other parasites that stimulate IgE antibodies of diverse specificities, thus precluding harmful hypersensitive reactions. Pre-clinical studies in healthy volunteers must be performed before any positive or negative conclusion can be drawn regarding cysteine peptidase-based vaccine implementation in humans.

## Conflict of Interest Statement

The authors declare that the research was conducted in the absence of any commercial or financial relationships that could be construed as a potential conflict of interest.
